# An Investigation of the Antiviral Potential of Phytocompounds against Avian Infectious Bronchitis Virus through Template-Based Molecular Docking and Molecular Dynamics Simulation Analysis

**DOI:** 10.3390/v15040847

**Published:** 2023-03-26

**Authors:** Irfan Gul, Amreena Hassan, Ehtishamul Haq, Syed Mudasir Ahmad, Riaz Ahmad Shah, Nazir Ahmad Ganai, Naveed Anjum Chikan, Mohamed Faizal Abdul-Careem, Nadeem Shabir

**Affiliations:** 1 Division of Animal Biotechnology, Faculty of Veterinary Sciences and Animal Husbandry, Shuhama, Sher-e-Kashmir University of Agricultural Sciences and Technology of Kashmir, Srinagar 190006, India; irfangul10@gmail.com (I.G.);; 2 Department of Biotechnology, University of Kashmir, Srinagar 190006, India; 3 Division of Computational Biology, Daskdan Innovations, Pvt. Ltd., Kashmir 190006, India; 4 Faculty of Veterinary Medicine, University of Calgary, 3330 Hospital Drive NW, Calgary, AB T2N 4N1, Canada

**Keywords:** infectious bronchitis virus, natural antiviral, pharmacokinetic, molecular docking, molecular dynamics simulation

## Abstract

Vaccination is widely used to control Infectious Bronchitis in poultry; however, the limited cross-protection and safety issues associated with these vaccines can lead to vaccination failures. Keeping these limitations in mind, the current study explored the antiviral potential of phytocompounds against the Infectious Bronchitis virus using in silico approaches. A total of 1300 phytocompounds derived from fourteen botanicals were screened for their potential ability to inhibit the main protease, papain-like protease or RNA-dependent RNA–polymerase of the virus. The study identified Methyl Rosmarinate, Cianidanol, Royleanone, and 6,7-Dehydroroyleanone as dual-target inhibitors against any two of the key proteins. At the same time, 7-alpha-Acetoxyroyleanone from *Rosmarinus officinalis* was found to be a multi-target protein inhibitor against all three proteins. The potential multi-target inhibitor was subjected to molecular dynamics simulations to assess the stability of the protein–ligand complexes along with the corresponding reference ligands. The findings specified stable interactions of 7-alpha-Acetoxyroyleanone with the protein targets. The results based on the in silico study indicate that the phytocompounds can potentially inhibit the essential proteins of the Infectious Bronchitis virus; however, in vitro and in vivo studies are required for validation. Nevertheless, this study is a significant step in exploring the use of botanicals in feed to control Infectious Bronchitis infections in poultry.

## 1. Introduction

Infectious Bronchitis (IB) is a highly contagious disease with significant economic implications for the global chicken industry. First documented in 1931, IB is mainly linked to respiratory, reproductive, digestive, and renal disorders in domestic chickens and various avian species [1,2]. Infectious bronchitis virus (IBV) replicates primarily in the epithelial cells of the respiratory tract, resulting in respiratory problems [3]. The epithelial cells in the oviduct and the kidney are also susceptible to IBV infection, impairing the quality and production of eggs and causing nephritis. IB infections can cause a mortality rate of 20–30% [4,5], increasing significantly with secondary infections in infected flocks [6,7]. The IBV is an enveloped positive-sense single-stranded RNA virus (+ssRNA) that belongs to the genus Gammacoronavirus of the *Coronaviridae* family [8]. The IBV RNA genome is non-segmented, about 27.6 kilobases in size, with a 5′ cap structure and 3′ poly-A tail resembling eukaryotic messenger RNAs. The genome also has a 3′ and 5′ untranslated regions (UTRs) [9,10,11]. About two-thirds of the IBV genome constituting Gene 1 is structured as two overlapping open reading frames (ORFs) translated into polyproteins 1a and 1ab due to ribosomal frameshift. Post-translational cleavage of the polyproteins 1a and 1ab results in the production of 15 non-structural proteins (NSPs). These NSPs include a main protease (Mpro), also known as 3C-like protease, a papain-like protease (PLpro), an RNA-dependent RNA-polymerase (RdRp), and other non-structural proteins [12]. The remainder of the genome codes for the primary structural proteins (spike, nucleocapsid, membrane and envelope) and the accessory proteins [13,14].

The management of IB is critical for ensuring the protection of animal welfare as well as global food security. The IBV vaccination regime is currently based mainly on the use of live attenuated vaccines (LAV) and inactivated vaccines. The vaccines provide robust protection against the IBV; however, LAV and inactivated vaccines often offer short-lived and modest cross-protection due to the considerable diversity in the prevalent IBV strains [15,16]. In addition, the attenuation mechanism of LAV is poorly understood, and the attenuation process is highly inefficient, both of which contribute to the inefficiencies that could lead to the LAVs regaining their virulence. Even though efforts are being made to develop effective vaccines against IBV, not much progress has been made in developing alternative control measures that can be used to keep IB in chickens under control [17]. There are significant problems associated with the widespread use of antibiotics in chickens [18]. Since IBV infection follows secondary bacterial infections [19,20], heavy use of antibiotics to contain secondary bacterial infections has led to the development of antibiotic resistance in various bacterial strains, and they decrease the microbiota in the gut, which destroys the ability of the chicken gastrointestinal system to absorb nutrients [21,22]. Recent research has demonstrated that including botanicals in chicken feed can improve the animal’s overall performance as well as their health, welfare, and production [23,24,25,26]. Botanicals might directly boost immunity and alter the intestinal microbiota composition to improve the body’s natural defense against infectious diseases [27,28,29,30,31,32]. Therefore, due to limited access to effective IBV vaccination, antibiotic-associated problems and the emergence of virulent IBV variants, there is a pressing need to research and develop alternative sustainable drugs that can lessen the impact of IBV infections in poultry.

As an alternative, antiviral herbs that do not pose any adverse health effects on poultry or humans are being considered as a potential solution [33]. The development of novel and alternative therapeutic agents has the potential to play a significant role in managing infectious diseases. A range of bio-derivatives and feed additives, such as plant extracts, prebiotics, probiotics, enzymes, and yeast, have previously demonstrated immunomodulatory properties, improving metabolic status, reducing stress levels, decreasing cytokine release by macrophages, and increasing antimicrobial activity, which can lead to an improved response to viral infections and reduced risk of associated side effects [33]. Despite the limited research on the antiviral properties of plants compared to their antimicrobial properties, several studies have demonstrated the potential of plant-derived antiviral substances against IBV. Specifically, extracts from various plants, such as *Thymus vulgaris*, *Mentha piperita*, and *Desmodium canadense*, have been shown to have antiviral effects against IBV [34]. Moreover, *Hypericum perforatum* L, *Sambucus nigra* and garlic were also found to inhibit IBV infection [33,35]. Extracts of *Achyranthes aspera*, *Neuroda procumbens*, *Panicum antidotale*, *Ochthochloa compressa*, and *Suaeda fruticosa* have been reported to have antiviral effects against poultry viruses, with the extracts of *S. icolados* and *O. compressa* showing the best results [33]. Essential oils and botanical oleoresins have also shown positive effects on IBV in chicken, reducing the clinical manifestation, pathological lesion, and RNA of the virus [33].

In order for a virus to replicate, it requires specific proteins that have functional and structural roles. Since the malfunction of these proteins influences the viral replication and spread of infection, they are at the center of the emphasis when it comes to the design and exploration of antiviral drugs. The Mpro, PLpro and the RdRp of IBV are the three active viral proteins that function in the replication, transcription and assembly of the virus. Therefore, these proteins have the potential to be targeted in the development of effective antivirals. Mpro and PLpro are two viral proteases recognized for their ability to carry out the proteolytic maturation and cleavage of polyprotein 1a and 1ab into various NSPs, each of which plays a unique and vital role [10,36,37]. Given the essential role of both proteases, they become an attractive target for developing antivirals against coronaviruses [38,39,40]. In contrast, RdRp is a crucial enzyme required for viral replication and has been investigated as a potential therapeutic target for a number of viruses, including SARS-CoV-2 [41,42,43].

The objective of this study was to use a bioinformatics approach to evaluate the antiviral properties of fourteen botanicals known to enhance the growth rate, production, feed efficiency, health status, and immunological responses of poultry. Several of these botanicals, including common yarrow, garlic, turmeric, and borage, have a history of being characterized as antimicrobial in poultry [44,45,46,47,48,49,50,51,52,53]. In addition, studies have shown that peppermint and chicory can improve the growth performance of broiler chickens without negatively affecting their intestinal morphology. Chicory forage has been found to be a potentially beneficial feed ingredient for broiler chickens [54,55]. Peppermint promotes growth in young broilers, improves their performance and carcass features, and reduces mortality [54,56]. A mixture of oregano, rosemary, and volatile fennel oils has been shown to have potent antibacterial activity against coliform bacteria, improving feed efficiency and carcass quality in broiler chickens [54,56,57,58,59,60,61,62,63,64]. Additionally, lemon balm have been found to have various impacts on body weight and bone health in broiler chickens [59]. The immunomodulatory properties of black cumin have been demonstrated in broiler chickens, significantly affecting weight gain and FCR [60,61,62]. Garlic powder and tulsi leaf powder were observed to potentially serve as an alternative to conventional antimicrobials, improving the production efficiency and immune status of birds by enhancing the T-cell mediated immune response [47,48,49,50]. It has been demonstrated that sumac (*Rhus coriaria*) has the potential as a feed additive for broilers, benefiting those raised in stressful situations by improving their growth and feed efficiency [65,66]. Adding marjoram leaf powder to the broiler diet significantly improved FCR, productivity, and daily body weight growth without affecting immune response [46].

As a first step for the study, we screened the selected botanicals based on ADMET-predicted pharmacokinetics and toxicity, which is followed by the template-based molecular docking of phytocompounds of the selected botanicals against Mpro, PLpro, and RdRp from IBV. The findings were further evaluated using molecular dynamics simulation analysis. In order to generate a comparative study, target viral proteins with native ligand inhibitors were selected as reference complexes.

## 2. Materials and Methods

This study performed molecular dynamics simulation using the Schrödinger software package version 2022-1 [67] and primary docking using Cresset Flare version 6. A SWISS-MODEL was used for homology modelling to generate a 3D model [68]. The PyMOL (https://pymol.org/, accessed on 25 January 2023) and LigPlot+ were used to visualize and generate binding poses [69].

### 2.1. Ligand Preparation

Botanical plants were collected through a literature survey, and their compounds were sourced from a curated database IMMPAT [70]. A local compound library named PhytoChemiome Library Version 1 (PCLibVer1) was compiled of about 1300 botanical compound ligands, which were retrieved in SDF format from the PubChem database (https://pubchem.ncbi.nlm.nih.gov/, accessed on 13 January 2023) [71]. Cresset Flare software was used for the preparation and docking of the ligands [72,73,74].

### 2.2. Protein Preparation

The 3-dimensional protein structures of IBV Mpro (PDB ID: 2Q6F) and PLpro (PDB ID: 4X2Z) were retrieved from RCSB Protein Data Bank database (https://www.rcsb.org/, accessed on 13 January 2023) [15,75,76]. The structure of Mpro was in complex with a Michael acceptor inhibitor (N3), which was removed from the structure. Since the crystal structure of IBV-RdRp was unavailable, protein homology modeling was performed using SWISS-MODEL based on the SARS-CoV2 RdRp structure (PDB ID: 7DFG) with 63.06% sequence identity [68]. The sequence of IBV-RdRp was obtained from NCBI reference sequence accession no. Np_066134.1. The homology model was subjected to structural refinement using the GalaxyRefine server (http://galaxy.seoklab.org/cgi-bin/submit.cgi?type=REFINE, accessed on 20 January 2023) [77]. The refined tertiary structure was analyzed by the PDBSum (http://www.ebi.ac.uk/thornton-srv/databases/pdbsum/, accessed on 20 January 2023) online server [78,79] (Figure S1). Subsequently, the water molecules and heteroatoms from all the structures were removed, and the proteins were subjected to energy minimization.

### 2.3. Determining the Target Sites

The binding sites of Michael acceptor inhibitor N3 was determined from the crystal structure of Mpro (2Q6F) using the inbuild utilities of Flare [15]. Myricetin, a known inhibitor of PLpro, was docked into the catalytic triad active site of PLpro [80,81]. The interacting residues were identified and designated as a ‘myricetin binding site’. The AT-9010 binding site of SARS-CoV-2 RdRp (PDB ID: 7ED5) was determined using Flare [82]. The site was then aligned with the IBV RdRp to identify the binding residues. The binding sites of Michael acceptor N3, myricetin and AT-9010 were selected as the target sites, while Michael acceptor inhibitor N3 (PubChem CID: 42627499), Myricetin (PubChem CID: 5281672) and AT-9010 (PubChem CID: 162642756) were taken as reference molecules for Mpro, PLpro and RdRp, respectively.

### 2.4. Pharmacokinetic Assessment

The potential biological properties of PCLibVer1 compounds were investigated using ADMETlab 2.0 (https://admetmesh.scbdd.com/, accessed on 17 January 2023) [83]. The server was used to estimate various properties related to pharmacokinetics and toxicity, such as solubility and permeability based on Lipinski’s Rule of Five, potential for drug-induced liver injury, and the likelihood of carcinogenicity. These predictions can provide valuable information on the potential biological activity and safety of the phytocompound when used in poultry, which are food animals. The screening criteria for ADMET properties were set to stringent standards: no violations of Lipinski’s Rule of Five, negative results for potential hepatotoxicity and carcinogenicity.

### 2.5. Virtual Screening

The screening of the PCLibVer1 compounds targeting IBV Mpro, PLpro and RdRp was performed using Cresset Flare software. The template-based docking of the ligands was performed sequentially using the Virtual screening protocol of Flare followed by Normal Docking, Accurate Docking, Extra Precision Docking (five runs) andExtra Precision Docking (ten runs) Protocol. At each stage, only half of the ligands were selected based on Gibbs free energy (ΔG) of the binding poses compared to the reference. A final list of ten ligands was generated against each target protein, wherein the ligands were compared, and a common botanical inhibitor against the target proteins was selected. Using the protein–ligand interaction tool of Schrödinger Maestro, post-docking investigations were performed, revealing the sizes and positions of binding sites, hydrogen-bond interactions, hydrophobic interactions, and bonding distances [67]. The common compound was evaluated for inhibitory binding with the target proteins via molecular dynamics simulation. The ligand’s best and most energetically optimal conformations were selected for molecular dynamics.

### 2.6. Molecular Dynamics

Molecular dynamics (MD) simulation was carried out with the Schrödinger software package. To begin, the protein–ligand complex was prepared using the “protein preparation wizard” feature in Schrödinger Maestro v2022.1. By employing the OPLS3 force field, the pre-processing steps for the protein preparation, including the addition of hydrogen atoms, the assignment of bond order, the removal of water molecules beyond 5.0 Å, and the establishment of a pH value of 7 were performed. Following the pre-processing, a solvated system according to the TIP3P water model was generated using the Desmond System Builder tool, wherein a cubic simulation box having periodic boundary conditions of 10 × 10 × 10 Å was prepared. Adding Cl or Na+ ions neutralized the system, and 0.15 M salts created an isosmotic state. The MD simulation was run using the Desmond molecular dynamics tool at constant pressure (1.013 bar) and temperature (300 K) for a period of 100 ns, with 1000 frames selected for the trajectory [84]. The post-MD analyses, including root mean square deviation (RMSD), root mean square fluctuations (RMSF), and protein–ligand contacts, were analyzed using the simulated interaction diagram tool.

## 3. Results

A comprehensive evaluation of the pharmacological properties of PCLibVer1 phytochemicals was performed using the ADMETLab 2.0. The screening criteria were used to select compounds that met specific standards for their ADMET properties, such as solubility, permeability, and potential toxicity. After the screening, 520 phytochemicals were identified as suitable for further analysis. These compounds were then prepared for molecular docking with targeted proteins using the Cresset Flare software.

### 3.1. Virtual Screening of Ligand Libraries

The screening of a set of phytocompounds of PCLibVer1 was conducted against the protein targets using a molecular docking protocol embedded in Flare software. The docking involves predicting how well the compounds bind to the proteins by estimating the strength of the chemical interactions between them. The docking process was carried out in several stages, starting with a basic docking procedure and increasing precision with each subsequent step. In the first phase, the Quick Docking protocol of Flare was employed, which proceeded with Normal Docking, Accurate Docking, Very Accurate Docking, Extra Precision Docking with five runs, and finally, Extra Precision Docking with ten runs. At each stage, the phytocompounds were ranked based on their ΔG binding score, and the top 50% of the phytocompounds with the lowest (more negative) scores were selected. Consequently, a list of the top ten hits (Table S2) was generated for each target protein, and these hits were compared to identify common inhibitor compounds (Table 1). Based on the comparison, several common hits were identified against the protein targets. Specifically, four phytocompounds were found to inhibit the proteins Mpro and PLpro, three molecules inhibited both Plpro and RdRp, and two molecules inhibited both Mpro and RdRp. Out of all the top phytocompounds, only phytocompound 2751796 was predicted to bind effectively to all three protein targets. In order to assess the effectiveness, the phytocompounds were compared to the reference molecules based on the ΔG score. This score reflects the binding affinity of a ligand to a protein and can help predict the potential inhibitory activity of a compound. The analysis revealed that each ligand had a greater or comparable negative ΔG score compared to the reference molecules, indicating a stronger binding affinity with the target protein.

### 3.2. Common Phytocompound Inhibitor against Mpro and Plpro

**Figure 1:**
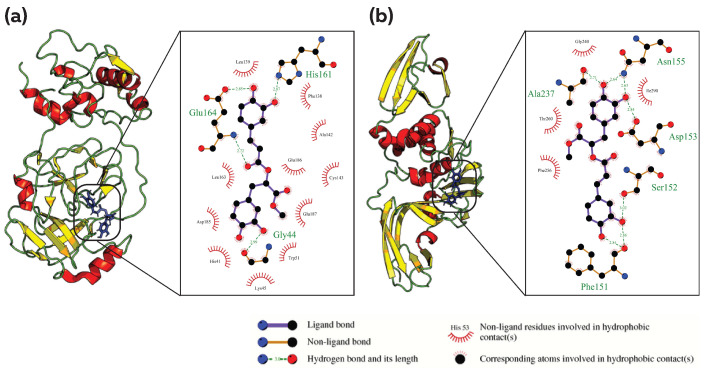
Analysis of protein–ligand interaction for Methyl rosmarinate (PubChem CID: 6479915) against Mpro and PLpro; Analysis of the binding pocket and types of interaction between (**a**) 6479915 and Mpro, (**b**) 6479915 and Plpro. The figure illustrates the 3D and 2D binding modes of Methyl rosmarinate with the key amino acid residues of the Mpro and Plpro.

The binding of phytocompounds to the IBV Mpro and Plpro proteins revealed four common hits. Of these, phytocompound 6479915 (Methyl rosmarinate) from *Mentha piperita* had the strongest binding to Mpro with a binding energy of −10.135 kcal/mol. The second most efficient binding was seen for phytocompound 23243692 (7-O-Methylrosmanol), which was followed by 23243694 (Epirosmanol), and 46883407 (Rosmaquinone β) with a binding energy of −8.966 kcal/mol, −8.945 kcal/mol and −8.759 kcal/mol, respectively. All of these phytocompounds are derived from the *Rosmarinus officinalis* botanical. When evaluating the binding of these phytocompounds to the PLpro protein, 23243692 showed the highest binding energy of −8.864 kcal/mol, which was followed by 23243694, 6479915 and 46883407 with a binding energy of −8.773 kcal/mol, −8.688 kcal/mol and −8.49 kcal/mol, respectively. The analysis of molecular interactions between phytocompounds and protein targets showed that phytocompound 6479915 formed hydrogen bonds with GLY44, HIS161 and GLU164 of Mpro, and PHE151, SER152, ASP153, ASN155 and ALA237 of PLpro (Figure 1a,b). Phytocompounds 23243692, 23243694, and 46883407 sharing similar structures showed only hydrophobic interactions with binding site amino acid residues of Mpro and PLpro, except for 46883407 forming two hydrogen bonds with ASN155 of PLpro (Figure S2b–d). All the ligands, however, showed non-bonded contacts, including van der Waals interactions and weak electrostatic interactions, that could contribute to the specificity of the protein–ligand interaction. A comparison of the phytocompounds binding to their corresponding target sites revealed common interacting residues for both proteins. These residues include HIS41, GLY44, LYS45, TRP51, CYS143, LEU163, ASP185, GLY 186 and GLU187 for Mpro. Similarly, for PLpro, the common interacting residues identified were PHE151, SER152, ASP153, ASN155, PHE256, ALA237, GLY240, THR260 and ILE290.

### 3.3. Common Phytocompound Inhibitor against Mpro and RdRp

**Figure 2:**
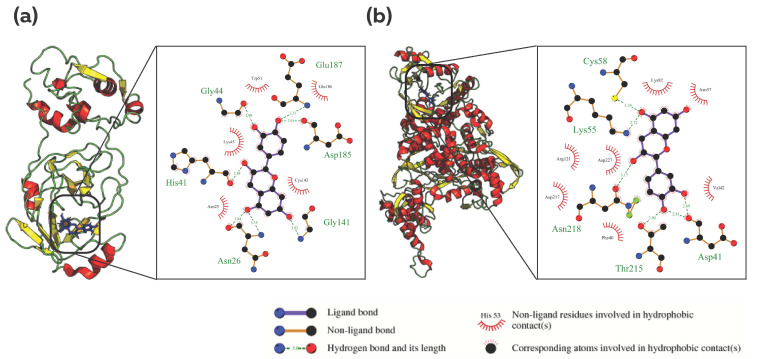
Analysis of protein–ligand interaction for Cianidanol (PubChem CID: 9064) against Mpro and RdRp; Analysis of the binding pocket and types of interaction between (**a**) 9064 and Mpro, (**b**) 9064 and RdRp. The figure illustrates the 3D and 2D binding modes of Cianidanol with the key amino acid residues of Mpro and RdRp.

The binding affinities of the two common phytocompounds were evaluated against the protein targets Mpro and RdRp. The findings revealed that the phytocompound 9064 (Cianidanol) exhibited binding energy of −9.497 kcal/mol and −9.389 kcal/mol against Mpro and RdRp, respectively, while the 13820511 (Isorosmanol) phytocompound exhibited binding energy of −9.191 kcal/mol and −8.154 kcal/mol against Mpro and Plpro, respectively. The analysis of molecular interactions between the phytocompounds and the protein targets revealed that the phytocompound 9064 formed hydrogen bonds with amino acid residues ASN26, HIS41, GLY44, GLY141, GLU187 and ASP185 of Mpro as well as with residues ASP41, LYS55, CYS58, THR215 and ASN218 of RdRp (Figure 2a,b). The phytocompound 13820511, on the other hand, formed hydrogen bonds with the amino acid residue HIS41 and LYS45 of the Mpro protein, and it showed only hydrophobic interactions with the binding site amino acid residues of the RdRp protein (Figure S3b). A comparison of the phytocompounds binding to their corresponding target sites revealed common interacting residues for both proteins. The analysis showed that for Mpro, both phytocompounds interacted with the amino acid residues ASN25, ASN26, HIS41, GLY44, LYS45, CYS143, ASP185, GLU186 and GLU187. Similarly, For RdRp, the common residues were VAL42, ASP41, PHE40, THR215, ASP217 and ASP227 (Figure S3c).

### 3.4. Common Phytocompound Inhibitor against PLpro and RdRp

**Figure 3:**
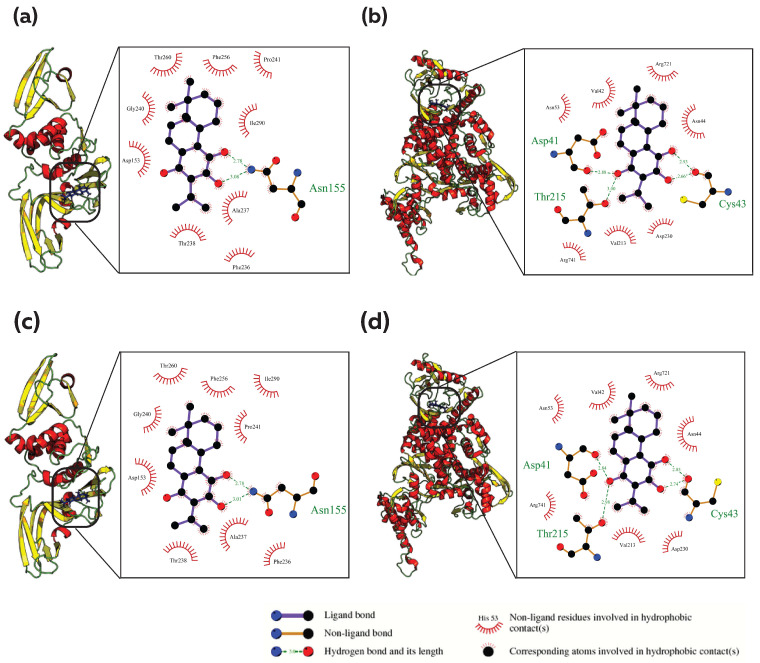
Analysis of protein–ligand interaction for Royleanone (PubChem CID: 442084) and 6,7-Dehydroroyleanone (PubChem CID: 2751794) against PLpro and RdRp; Analysis of the binding pocket and types of interaction between (**a**) 442084 and PLpro, (**b**) 2751794 and RdRp, (**c**) 2751794 and PLpro, (**d**) 442084 and RdRp. The figure illustrates the 3D and 2D binding modes of Royleanone and 6,7-Dehydroroyleanone with the key amino acid residues of the PLpro and RdRp.

The phytocompounds 442084 (Royleanone), 2751794 (6,7-Dehydroroyleanone), and 75552 (Diallyl tetrasulfide) were found to be common inhibitors against the viral PLpro and RdRp enzyme targets. The phytocompound 442084 exhibited binding energies of −8.794 kcal/mol and −7.961 kcal/mol against PLpro and RdRp, respectively, while the phytocompound 2751794 showed binding energies of −8.28 kcal/mol and −8.117 kcal/mol against PLpro and RdRp. The phytocompound 75552 exhibited binding energies of −7.916 and −8.077 kcal/mol against PLpro and RdRp, respectively. These binding energies were more substantial compared to the reference compounds. Analysis of molecular interactions revealed that the phytocompounds 442084 and 2751794 formed hydrogen bonds with the amino acid residues ASN155 of PLpro and ASP41, CYS43 and THR215 of RdRp (Figure 3a–d). The phytocompound 75552 showed hydrophobic interactions with the binding site amino acid residues of both PLpro and RdRp along with 41 and 50 non-bonding interactions, which may include Vander Waals interactions and weak electrostatic interactions (Figure S4c). Comparing the binding of the phytocompounds to each protein showed common interacting residues, including ASP153, ASN155, PHE236, ALA237, THR238, PHE256 and ILE290 for PLpro and ASP41, VAL42, CYS43, ASP230, THR215 and ARG741 for RdRp (Figure S4d).

### 3.5. Common Phytocompound Inhibitor against Mpro, PLpro and RdRp

**Figure 4:**
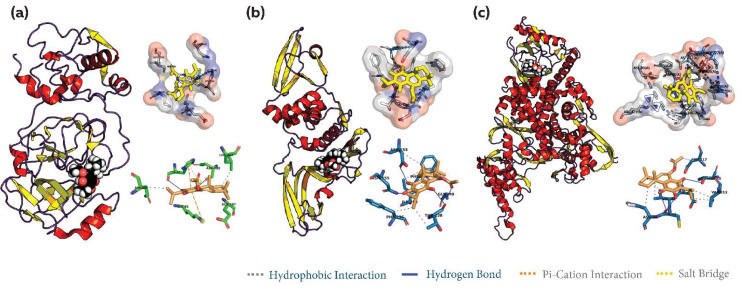
Analysis of the binding of the screened phytocompound hit (2751796) to the protein targets of the virus. The figure illustrates the binding and interaction analysis of (**a**) 2751796-Mpro, (**b**) 2751796-PLpro, and (**c**) 2751796-RdRp, depicting various types of interactions between the ligand and the protein targets.

The phytocompound 2751796 (7alpha-Acetoxyroyleanone) from the Rosmarinus officinalis botanical exhibited strong binding affinity against all the IBV enzyme targets. The binding affinity of the phytocompound against Mpro was found to be −8.608 kcal/mol, which was comparable to that of the reference inhibitor (−9.698 kcal/mol). Additionally, the phytocompound exhibited a binding affinity of −8.703 kcal/mol and −8.9 kcal/mol against PLpro and RdRp, respectively. These values are higher than those of the corresponding reference compounds, −7.672 kcal/mol and −2.265 kcal/mol, respectively. These results indicate the multi-target inhibitory potential of the phytocompound 2751796 against these enzymes. Furthermore, the analysis of the molecular interactions revealed the presence of specific interactions between the phytocompound 2751796 and the target proteins. These interactions include hydrogen bonds, salt bridges, pi–cation and hydrophobic interactions. Specifically, the phytocompound formed two hydrogen bonds with the amino acids HIS41 and ASP185, a salt bridge with HIS41, pi–cation interactions with HIS41, LYS45, and hydrophobic interactions with the ASN25, LEU163 and GLU187 amino acid residues of Mpro (Figure 4a). The phytocompound formed four hydrogen bonds with the amino acids ASP153, ASN155, THR238 and GLY240 and exhibited hydrophobic interactions with ASP153, PHE236, THR238, PHE256 and ILE290 of PLpro (Figure 4b). In the case of RdRp, the phytocompound formed four hydrogen bonds with ASP41, CYS43, CYS43 and THR215 and exhibited hydrophobic interactions with VAL42, ASN44, VAL213, and ASP217 (Figure 4c).

### 3.6. Molecular Dynamics Analysis

**Figure 5:**
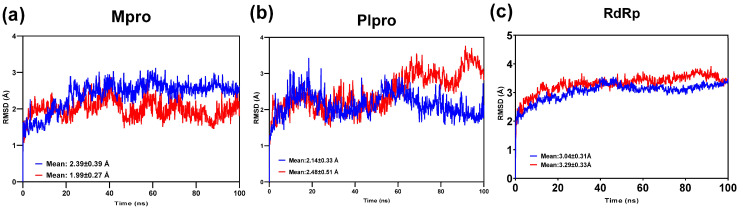
RMSD trajectories of ligand-bound proteins analyzed relative to C-α atoms over the 100 ns MD simulation. The RMSD trajectories for the (**a**) Ligand-bound Mpro protein, (**b**) Ligand-bound PLpro protein, and (**c**) Ligand-bound RdRp protein. The RMSD values for the 2751796 phytocompound and the respective reference compounds are illustrated in red and blue colors, respectively.

The root mean square deviation (RMSD) values of the Mpro-2751796, PLpro-2751796, and RdRp-2751796 complexes were evaluated throughout the 100 ns molecular dynamics (MD) simulation. These RMSD values were used to determine the equilibration of the protein C-alpha atoms for each complex compared to their respective reference complexes. The findings revealed that the equilibration of the protein C-alpha atoms for Mpro-2751796 occurred at an RMSD value of 1.99 ± 0.27 Å, which was lower than the Mpro-N3 reference complex (2.39 ± 0.39 Å) (Figure 5a). Likewise, the equilibration of the protein C-alpha atoms for PLpro-2751796 occurred at an RMSD value of 2.48 ± 0.51 Å, which was higher than the PLpro-myricetin reference complex (2.14 ± 0.33 Å). The phytocompound displayed comparable binding to the PLpro up to 60ns but showed higher fluctuations from 60 to 100 ns (Figure 5b). The equilibration of the protein C-alpha atoms for RdRp-2751796 occurred at an RMSD value of 3.29 ± 0.33 Å, which was slightly higher than the RdRp- AT9010 reference complex (3.04 ± 0.31 Å) (Figure 5c). These results provide valuable information about the structural stability of these complexes and suggest that the 2751796 phytocompound has a relatively stable residence in the binding pocket of the target proteins at the given docking pose.

**Figure 6:**
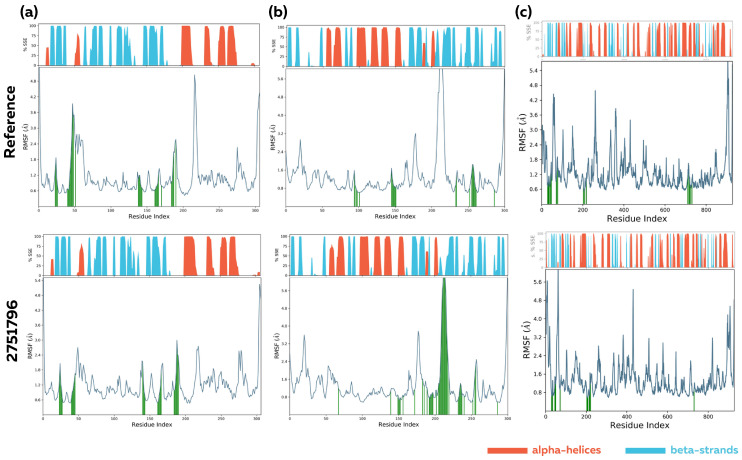
Analysis of RMSF trajectories and secondary structure elements (SSE) distribution over the 100 ns MD simulation for (**a**) Ligand–Mpro, (**b**) Ligand–PLpro, and (**c**) Ligand–RdRp complexes. Protein residues that interact with the ligand are marked with green-colored vertical bars. The alpha-helices and beta-strands SSEs are shown in red and blue colors, respectively.

Upon examining the residue-based root mean square fluctuation (RMSF) for each investigated complex, no significant differences were observed. However, the binding of the 2751796 phytocompound to Mpro caused a slight loss of mobility (Figure 6a). In contrast, binding the phytocompound to PLpro and RdRp did not cause any significant change in the flexibility of the amino acid residues compared to the binding of the reference compounds (Figure 6b,c). These results are consistent with the analysis of secondary structure element (SSE) distribution, which showed no significant change in the secondary structure of the protein upon binding of the phytocompound compared to the reference compounds.

**Figure 7:**
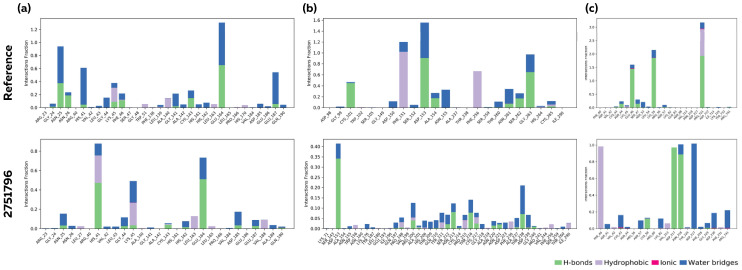
The protein–ligand interaction diagram illustrating the fraction of amino acids interacting with the ligands monitored over the 100 ns MD simulation. Analysis of protein–ligand interaction for (**a**) Ligand–Mpro, (**b**) Ligand–PLpro, and (**c**) Ligand–RdRp complexes. The protein–ligand interactions are illustrated as hydrogen bonds, hydrophobic, ionic, and water bridges. The stacked bar charts are normalized over the course of the trajectory.

During the 100 ns MD simulation, hydrogen bonds, hydrophobic interactions including pi–cation, pi–pi stacking, water bridges and ionic interactions formed by the ligand with the amino acids of the proteins were analyzed. The phytocompound bound comparably to the reference compounds in the case of Mpro, as indicated by the number of amino acid contacts and the retention of the interaction throughout the simulation time (measured as interaction fractions) (Figure 7a). On the other hand, in the case of PLpro and RdRp, the phytocompound interacted with a greater number of amino acid residues than the reference compounds, indicating a stronger binding of the ligand to those amino acids (Figure 7b,c). Although the reference compounds interacted with fewer residues, the binding was observed over a larger portion of the trajectory, indicating consistent binding. Notably, ionic interactions were absent in the protein–reference and protein–phytocompound complexes among the variety of interactions analyzed.

## 4. Discussion

Among poultry diseases, IB is a widespread disease affecting poultry globally, causing high morbidity rates and mortality along with decreased egg and meat production. Currently, the control of IBV is essentially attempted using live attenuated and inactivated vaccines. However, the efficacy of these vaccines is being limited by the increasing genetic diversity of IBV and the emergence of new IBV variants. In view of these difficulties, it is crucial to find new ways of controlling IB in chickens. This study aimed to investigate the antiviral effect of phytocompounds from fourteen botanicals against the replication of the IBV in poultry. These botanicals were selected based on previous research indicating their potential to improve poultry growth rate, feed-to-gain ratio, health status, and immune system function (Table S1). Initially, the study assessed the pharmacological properties of the phytochemicals and identified 520 phytocompounds that met specific standards for their ADMET properties. Molecular docking analysis revealed several common hits against IBV Mpro, PLpro and RdRp. Specifically, phytocompounds 6479915, 23243692, 23243694, and 46883407 were found to inhibit both Mpro and PLpro, while phytocompounds 9064 and 13820511 exhibited inhibition against Mpro and RdRp. Additionally, phytocompounds 442084, 2751794, and 75552 were identified as common inhibitors against the viral PLpro and RdRp enzyme targets. Of all the potential inhibitors, only Phytocompound 2751796 was predicted to bind effectively to all three protein targets.

The prediction of ADMET properties, such as solubility, permeability, liver injury potential, and carcinogenicity, plays a critical role in evaluating the biological activity and safety of phytocompounds. It is essential to consider these predictions to make informed decisions regarding the use of phytocompounds in poultry production and to ensure the health and well-being of these animals. The pharmacokinetic properties including the oral absorption, solubility, permeability and systemic distribution potential of the phytocompounds in poultry were determined by using Lipinski’s Rule of Five. This rule states that drugs with desirable pharmacokinetic properties typically have a molecular weight of less than 500 Da, a logP value (the octanol–water partition coefficient) of less than 5, no more than 10 hydrogen bond donors and no more than 5 hydrogen bond acceptors [85]. Additionally, prediction of potential toxicity was estimated to evaluate the likelihood of drug-induced liver injury and carcinogenicity. Drug-induced liver injury is a common adverse effect of many drugs and can have severe consequences for animal health. Carcinogenicity, on the other hand, is the potential of a substance to cause cancer and is an essential consideration for substances used in food animals.

The molecular docking study evaluated the binding energy and molecular interactions between the phytocompounds and target proteins and identified potential inhibitors. Among the evaluated inhibitors, Methyl Rosmarinate (PubChem CID: 6479915) was found to be an effective inhibitor of both Mpro and PLpro. Methyl Rosmarinate is a phytocompound derived from the *Mentha piperita* plant, which is commonly known as peppermint. It is a phenolic compound with antioxidant properties and has been studied for its potential health benefits. Methyl rosmarinate shows antioxidative and antifungal activities. It has inhibitory activities against tyrosinase, α-glucosidase, and matrix metalloproteinase-1 (MMP-1) [86,87,88,89,90]. The phytocompound Cianidanol (PubChem CID 9064) demonstrated potency as an inhibitor of Mpro and RdRp. Cianidanol is an antioxidant flavonoid in the *Ocimum sanctum* plant, which is also known as Holy basil or Tulsi. This plant is widely used in traditional medicine, particularly in India and other parts of Asia, due to its medicinal properties. Studies have shown that cianidanol has the ability to inhibit both the Mpro and spike proteins of SARS-CoV-2 as well as play a therapeutic and immunomodulatory role in chronic hepatitis [91,92,93]. At the same time, Royleanone (PubChem CID: 442084) and 6,7-Dehydroroyleanone (PubChem CID: 2751794) were identified as potent inhibitors of Plpro and RdRp. Another royleanone phytocompound, 7-alpha-Acetoxyroyleanone (PubChem CID: 2751796), was identified with multi-target inhibitory potential against all three IBV target proteins viz Mpro, Plpro and RdRp. The royleanones are a diterpenoids class of compounds found in the *Rosmarinus officinalis* plant, which is commonly known as rosemary. Studies have shown that these compounds have the potential to inhibit P-Glycoprotein, which may combat multidrug resistance (MDR) and induce cytotoxic effects in cancers [94,95].

The MD simulation studies demonstrate that 7-alpha-Acetoxyroyleanone exhibits stable binding to the binding pockets of the target protein as compared to known inhibitor reference compounds. Specifically, the RMSD analysis indicates the steady binding of 7-alpha-Acetoxyroyleanone to Mpro and RdRp throughout the MD simulation, and to Plpro for most of the simulation. The RMSF analysis characterizes protein backbone mobility and indicates the minimal effect of 7-alpha-Acetoxyroyleanone binding on the flexibility of target proteins, which was consistent with the secondary structure analysis. The findings also reveal various interactions, such as hydrogen bond interactions and non-bonded contacts, providing stability to the binding of 7-alpha-Acetoxyroyleanone to the target protein. These results validate that 7-alpha-Acetoxyroyleanone may serve as a multi-target inhibitor against Mpro, Plpro, and RdRp of IBV.

The research findings suggest that cianidanol, methyl rosmarinate, and royleanones hold promise as nutraceuticals for therapeutic purposes against the infectious bronchitis virus, potentially serving as a natural substitute for antibiotics. Additionally, the botanicals *Ocimum sanctum*, *Mentha piperita*, and *Rosmarinus officinalis* can be used as feed additives as a prophylactic strategy against the virus. In summary, using natural phytocompounds and botanicals in poultry farming shows great potential to combat IBV infection while ensuring the health and welfare of the poultry.

## Figures and Tables

**Table 1:** Comparison of top ligands to identify common inhibitor against the target proteins.

Pubchem CID	Mpro	Plpro	RdRp	Phytochemical Name	Botanical
Reference	−9.698	−7.672	−2.265	-	-
2751796	−8.608	−8.703	−8.9		
	
				7alpha-Acetoxyroyleanone	*Rosmarinus officinalis*
6479915	−10.135	−8.688	-		
				Methyl rosmarinate	*Mentha piperita*
23243692	−8.966	−8.864			
		
			-	7-O-Methylrosmanol	*Rosmarinus officinalis*
23243694	−8.945	−8.773			
			-	Epirosmanol	*Rosmarinus officinalis*
46883407	−8.759	−8.49			
			-	Rosmaquinone β	*Rosmarinus officinalis*
442084		−8.794	−7.961		
	-			Royleanone	*Rosmarinus officinalis*
2751794		−8.28	−8.117		
		
	-			6,7-Dehydroroyleanone	*Rosmarinus officinalis*
75552		−7.916	−8.077		
	-			Diallyl tetrasulfide	*Allium Sativum*
9064	−9.497	-	−9.389		
				Cianidanol	*Ocimum sanctum*
13820511	−9.191	-	−8.154		
				Isorosmanol	*Rosmarinus officinalis*

## Data Availability

The data presented in this study are openly available in the National Center for Biotechnology Information (NCBI) and Indian Medicinal Plants, Phytochemistry and its Therapeutics (IMPPAT).

## Supplementary Materials

The following supporting information can be downloaded at: https://www.mdpi.com/article/10.3390/v15040847/s1, Table S1: List of Botanicals included in the study: Table S2: List of top 10 hits against each target protein of IBV. The ligands are identified by their Pubchem Compound Identifier (CID);Figure S1: Analysis of IBV RdRp tertiary structure using PDBsum. (a) Secondary structure diagram of the protein, with pink arrows representing strands and purple springs representing helices, and various motifs depicted in red. (b) Assessment of refined protein structure through Ramachandran plot analysis, with core allowed regions in red, disallowed regions in brown, and regions with limited utilization in pale yellow; Figure S2:Analysis of protein-ligand interaction for common phytocompounds against Mpro and PLpro; Analysis of the binding pocket and the types of interaction between (a) 6479915-Mpro and 6479915- PLpro. (b) 23243692-Mpro and 23243692-PLpro. (c) 23243694-Mpro and 23243694-PLpro. (d) 46883407-Mpro and 46883407-PLpro. (e) A superposed plot of the protein bound to 6479915, 23243692, 23243694 and 46883407 phytocompounds. The circles and ellipses indicate common protein residues between protein-ligand complexes; Figure S3: Analysis of protein-ligand interaction for common phytocompounds against Mpro and RdRp; Analysis of the binding pocket and the types of interaction between (a) 9064-Mpro and 9064-RdRp. (b) 13820511-Mpro and 13820511-RdRp. (c) A superposed plot of the protein bound to phytocompounds 9064 and 13820511. The circles and ellipses indicate common protein residues between protein-ligand complexes; Figure S4: Analysis of protein-ligand interaction for common phytocompounds against PLpro and RdRp; Analysis of the binding pocket and the types of interaction between (a) 442084-PLpro and 442084-RdRp. (b) 2751794-PLpro and 2751794-RdRp. (c) 75552-PLpro and 75552-RdRp. (d) A superposed plot of the protein bound to 442084, 2751794 and 75552 phytocompounds. The circles and ellipses indicate common protein residues between protein-ligand complexes.
