# Supplementary material for: An Investigation of the Antiviral Potential of Phytocompounds against Avian Infectious Bronchitis Virus through Template-Based Molecular Docking and Molecular Dynamics Simulation Analysis
Source: Viruses. 2023 Mar 26;15(4):847. doi: 10.3390/v15040847 (PMC10144825; doi:10.3390/v15040847)
Supplement: Supplementary file 1 [file viruses-15-00847-s001.zip › viruses-2283779-supplementary.pdf]

Supplementary:

Table S1: List of Botanicals included in the study.

Botanical	Common Name	Reference
Achillea millefolium	Common Yarrow	[1-3]
Allium sativum	Garlic	[4-7]
Borago officinalis	Borage	[8, 9]
Cichorium intybus	Chicory	[10, 11]
Curcuma longa	Turmeric	[12]
Foeniculum vulgare	Fennel	[13, 14]
Melissa officinalis	Lemon balm	[15]
Mentha Piperita	Peppermint	[10, 16]
Nigella sativa	Black Cumin	[17-19]
Ocimum sanctum	Tulsi	[6]
Origanum majorana	Marjoram	[3]
Origanum Vulgare	Oregano	[20, 21]
Rhus coriaria	Sicilian sumac	[22, 23]
Rosmarinus officinalis	Rosemary	[1, 20, 24]

Table S2: List of top 10 hits against each target protein of IBV. The ligands are identified by their Pubchem Compound Identifier (CID).

IBV Mpro	IBV PLpro	IBV RdRp
6479915	6466	9064
9799386	23243692	2751796
9064	442084	9970764
13820511	23243694	73588
23243692	2751796	10475714
23243694	6479915	6424189
46883407	46883407	13820511
5281792	2751795	2751794
160142	2751794	75552
2751796	75552	442084

(a)

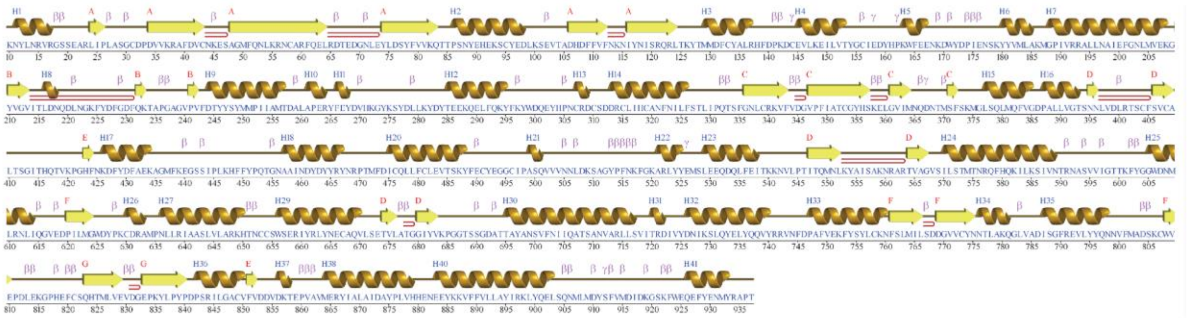

(b)

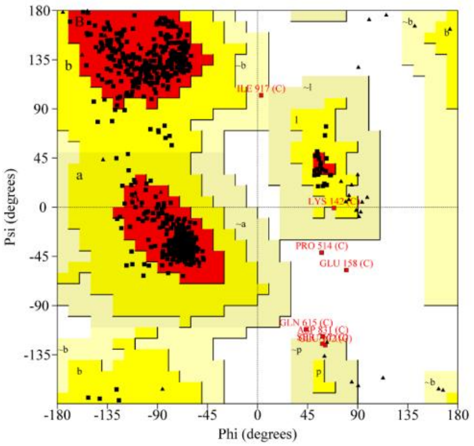

Plot statistics

Residues in most favoured regions [A,B,L]	790	93.8%
Residues in additional allowed regions [a,b,l,p]	45	5.3%
Residues in generously allowed regions [~a,~b,~l,~p]	4	0.5%
Residues in disallowed regions	3	0.4%
	----	------
Number of non-glycine and non-proline residues	842	100.0%
Number of end-residues (excl. Gly and Pro)	2	
Number of glycine residues (shown as triangles)	48	
Number of proline residues	36	

Total number of residues	928	

Figure S1: Analysis of IBV RdRp tertiary structure using PDBsum. (a) Secondary structure diagram of the protein, with pink arrows representing strands and purple springs representing helices, and various motifs depicted in red. (b) Assessment of refined protein structure through Ramachandran plot analysis, with core allowed regions in red, disallowed regions in brown, and regions with limited utilization in pale yellow.

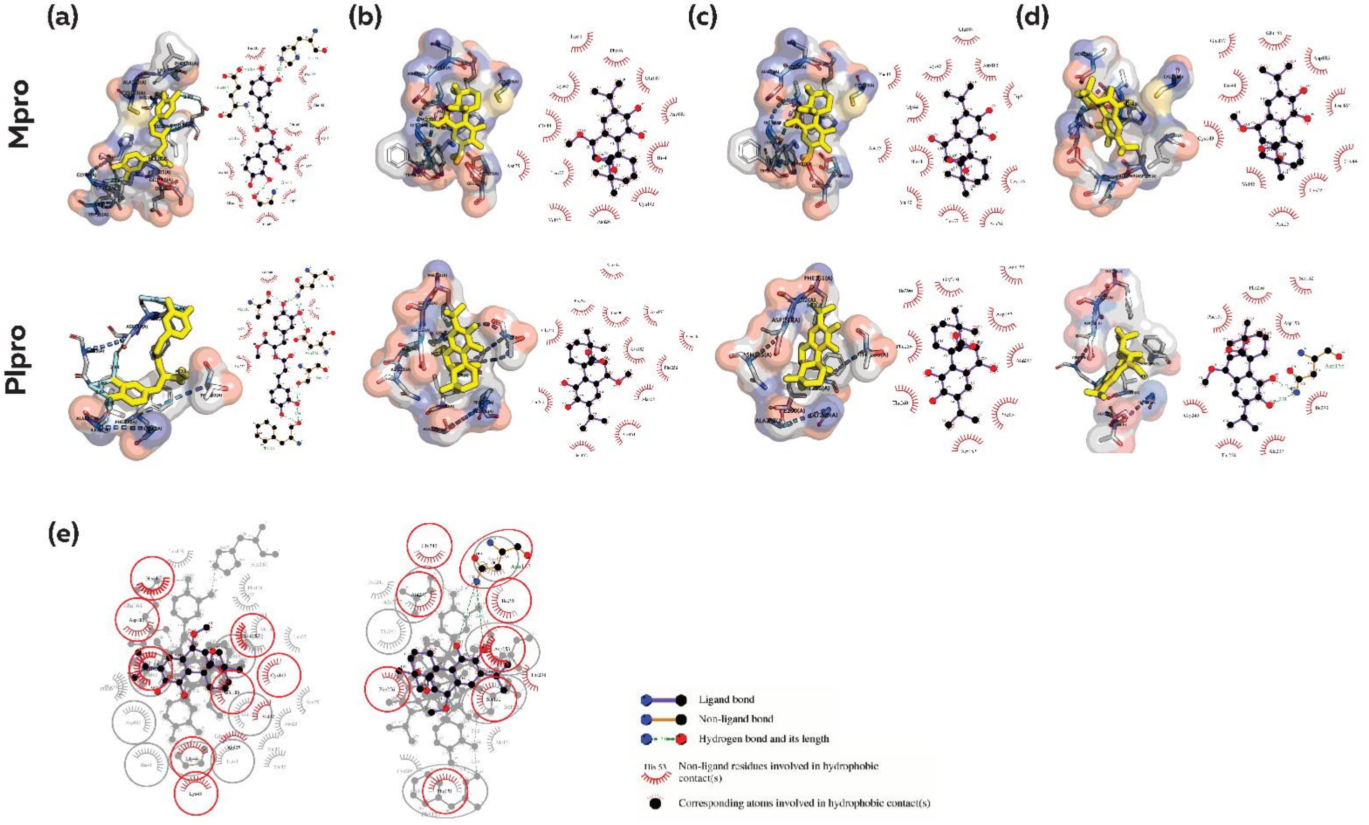

Figure S2. Analysis of protein-ligand interaction for common phytocompounds against Mpro and PLpro; Analysis of the binding pocket and the types of interaction between (a) 6479915-Mpro and 6479915-PLpro. (b) 23243692-Mpro and 23243692-PLpro. (c) 23243694-Mpro and 23243694-PLpro. (d) 46883407-Mpro and 46883407-PLpro. (e) A superposed plot of the protein bound to 6479915, 23243692, 23243694 and 46883407 phytocompounds. The circles and ellipses indicate common protein residues between protein-ligand complexes.

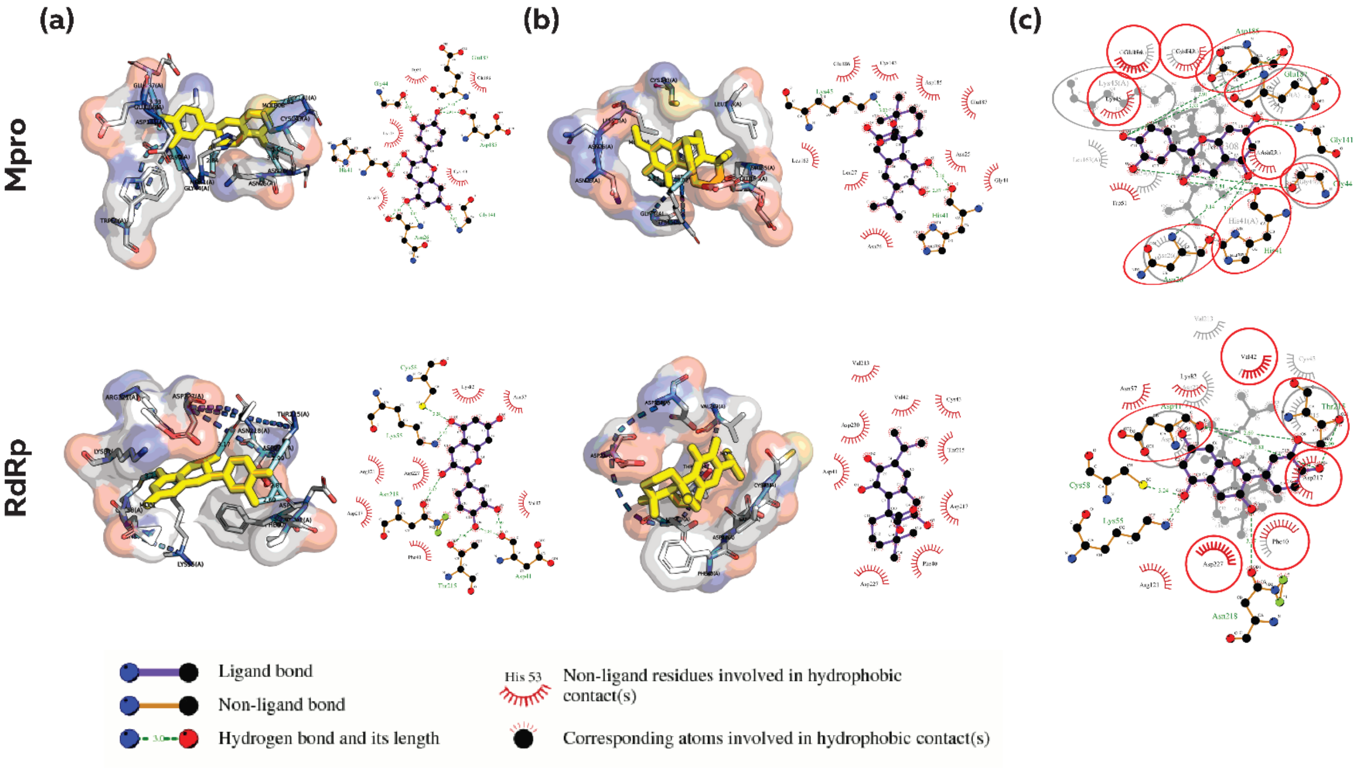

Figure S3. Analysis of protein-ligand interaction for common phytocompounds against Mpro and RdRp; Analysis of the binding pocket and the types of interaction between (a) 9064-Mpro and 9064-RdRp. (b) 13820511-Mpro and 13820511-RdRp. (c) A superposed plot of the protein bound to phytocompounds 9064 and 13820511. The circles and ellipses indicate common protein residues between protein-ligand complexes.

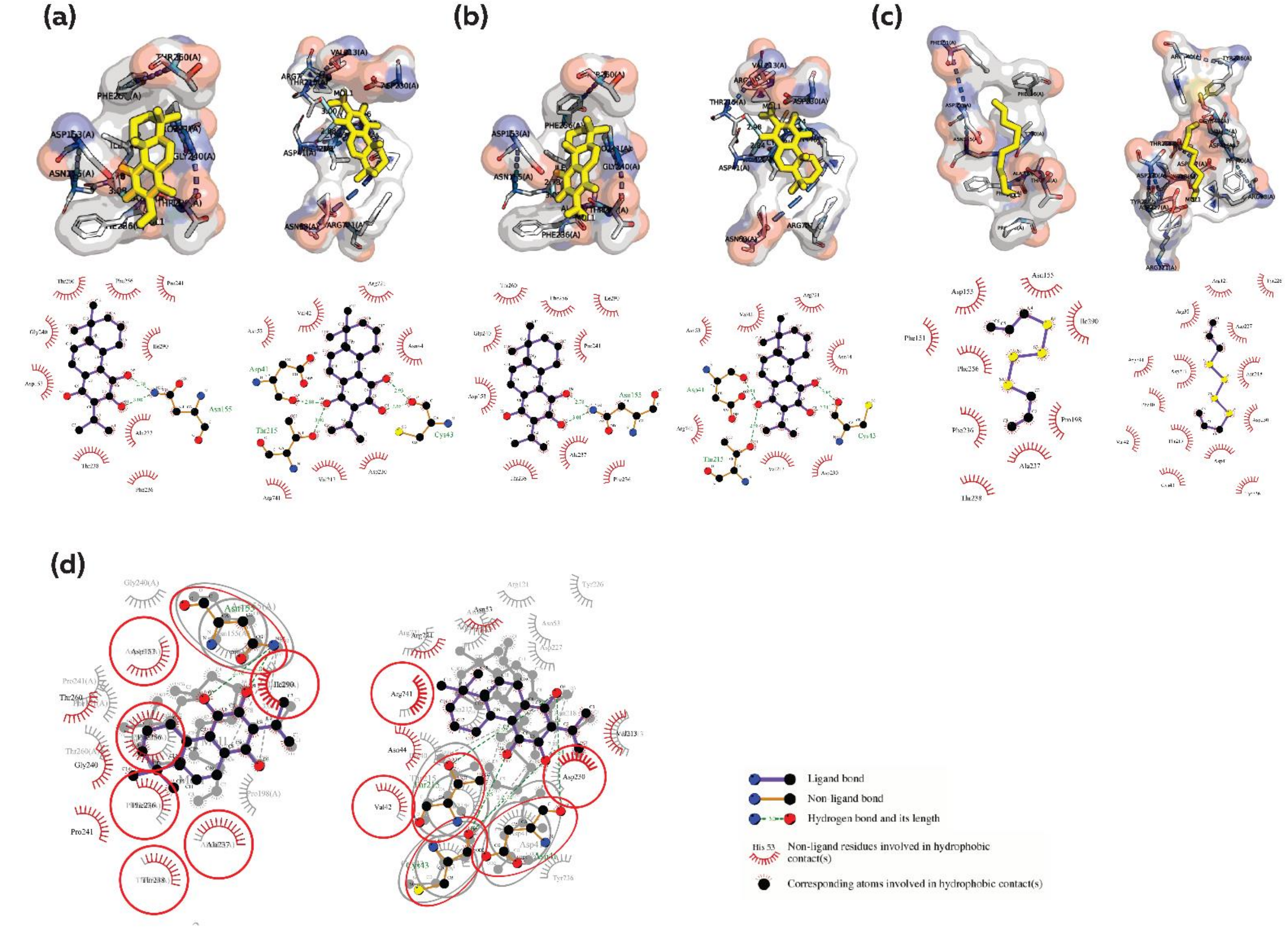

Figure S4. Analysis of protein-ligand interaction for common phytocompounds against PLpro and RdRp; Analysis of the binding pocket and the types of interaction between (a) 442084-PLpro and 442084-RdRp. (b) 2751794-PLpro and 2751794-RdRp. (c) 75552-PLpro and 75552-RdRp. (d) A superposed plot of the protein bound to 442084, 2751794 and 75552 phytocompounds. The circles and ellipses indicate common protein residues between protein-ligand complexes.

References:

1. Norouzi, B., et al., *Effect of Different Dietary Levels of Rosemary (Rosmarinus Officinalis) and Yarrow (Achillea Millefolium) on the Growth Performance, Carcass Traits and Ileal Micro-biota of Broilers.* Italian Journal of Animal Science, 2015. **14**(3): p. 3930.
2. Cross, D.E., R.M. McDevitt, and T. Acamovic, *Herbs, thyme essential oil and condensed tannin extracts as dietary supplements for broilers, and their effects on performance, digestibility, volatile fatty acids and organoleptic properties.* British Poultry Science, 2011. **52**(2): p. 227-237.
3. Cross, D.E., et al., *The effect of herbs and their associated essential oils on performance, dietary digestibility and gut microflora in chickens from 7 to 28 days of age.* British Poultry Science, 2007. **48**(4): p. 496-506.
4. Horton, G.M.J., M.J. Fennell, and B.M. Prasad, *Effect of dietary garlic (Allium sativum) on performance, carcass composition and blood chemistry changes in broiler chickens.* 1991. **71**(3): p. 939-942.
5. Pistova, V., H. ARPÁŠOVÁ, and C.J.J.o.C.E.A. HRNČÁR, *The effect of the humic acid and garlic (Allium sativum L.) on performance parameters and carcass characteristic of broiler chicken.* 2016.
6. Sheoran, N., et al., *Nutrigenomic evaluation of garlic (Allium sativum) and holy basil (Ocimum sanctum) leaf powder supplementation on growth performance and immune characteristics in broilers.* 2017. **10**(1): p. 121.
7. Ogunlesi, O., et al., *Effects of dietary garlic (Allium sativum) meal on skin thickness and fat deposition in commercial broiler chickens.* 2017. **20**(2): p. 118-24.
8. Ibrahim, D.K., K.A. Salman, and F.M.J.i.p.s.j. Al-Khilani, *Effect of supplementation aqueous extract of borage (Borago officinalis) and Melilotus (Melilotus officinalis) to drinking water on production performance of broiler during summer season.* 2016. **10**(1).
9. Boruta, A., J. Niemiec, and L. Marcin. *The effect of borage seeds in hens diet on fatty acids composition in egg yolk*. in *Proceedings of the XVII European Symposium on the Quality of Poultry Meat and XI European Symposium on the Quality of Eggs and Egg Products. Golden Tulip Parkhotel. Doorwerth, Netherlands*. 2005.
10. Poursina, B., et al., *Effect of peppermint (Mentha piperita L.), thyme (Thymus vulgaris L.) and chicory (Chicorium intybus L.) on performance and intestine morphology of broilers.* 2016. **31**(6).
11. Liu, H.Y., et al., *Growth performance, digestibility, and gut development of broiler chickens on diets with inclusion of chicory (Cichorium intybus L.).* Poultry Science, 2011. **90**(4): p. 815-823.
12. Mueller, K., et al., *Influence of broccoli extract and various essential oils on performance and expression of xenobiotic-and antioxidant enzymes in broiler chickens.* 2012. **108**(4): p. 588-602.
13. Cetin, E., et al., *The effect of volatile oil mixtures on the performance and ilio-caecal microflora of broiler chickens.* British Poultry Science, 2016. **57**(6): p. 780-787.
14. Mohammed, A.A. and R.J.J.I.J.o.P.S. Abbas, *The effect of using fennel seeds (Foeniculum vulgare L.) on productive performance of broiler chickens.* 2009. **8**(7): p. 642-644.
15. Kwiecieñ, M.g. and A.J.J.o.e. Winiarska-Mieczan, *Effect of addition of herbs on body weight and assessment of physical and chemical alterations in the tibia bones of broiler chickens.* 2009. **14**(4).
16. Ocak, N., et al., *Performance of broilers fed diets supplemented with dry peppermint (Mentha piperita L.) or thyme (Thymus vulgaris L.) leaves as growth promoter source.* 2008. **53**(4): p. 169.
17. Umar, S., et al., *Effects of Nigella sativa on immune responses and pathogenesis of avian influenza (H9N2) virus in turkeys.* Journal of Applied Poultry Research, 2016. **25**(1): p. 95.

18. Al-Hothaify, S. and M.J.A.J.o.R.C. Al-Sanabani, *The effects of supplementation Nigella sativa seeds as a natural substance on growth rate, some serum indices, carcass quality and antibody titers of broiler birds.* 2016. **4**(3): p. 43-51.
19. Guler, T., et al., *Effect of dietary supplemental black cumin seeds on antioxidant activity in broilers.* 2007. **63**(9): p. 1060-1063.
20. Franciosini, M.P., et al., *Effects of oregano (Origanum vulgare L.) and rosemary (Rosmarinus officinalis L.) aqueous extracts on broiler performance, immune function and intestinal microbial population.* Journal of Applied Animal Research, 2016. **44**(1): p. 474-479.
21. Ghazi, S., T. Amjadian, and S. Norouzi, *Single and combined effects of vitamin C and oregano essential oil in diet, on growth performance, and blood parameters of broiler chicks reared under heat stress condition.* International Journal of Biometeorology, 2015. **59**(8): p. 1019-1024.
22. Shariatmadari, F. and R. Shariatmadari, *Sumac (Rhus coriaria) supplementation in poultry diet.* World's Poultry Science Journal, 2020. **76**(2): p. 358-364.
23. Azizi, M., et al., *Effect of incremental levels of sumac (Rhus coriaria L.) seed powder on growth, carcass traits, blood parameters, immune system and selected ileal microorganisms of broilers.* Vet Ital, 2020. **56**(3): p. 185-192.
24. Ghazalah, A., A. El-Hakim, and A.J.E.P.S.J. Refaie, *Response of broiler chicks to some dietary growth promoters throughout different growth period.* 2007. **27**: p. 53-57.
